# A Stroll Through the History of Monoxenous Trypanosomatids Infection in Vertebrate Hosts

**DOI:** 10.3389/fcimb.2022.804707

**Published:** 2022-02-15

**Authors:** Carolina Boucinha, Valter Viana Andrade-Neto, Vítor Ennes-Vidal, Marta Helena Branquinha, André Luis Souza dos Santos, Eduardo Caio Torres-Santos, Claudia Masini d’Avila-Levy

**Affiliations:** ^1^ Instituto Oswaldo Cruz, Fundação Oswaldo Cruz (FIOCRUZ), Rio de Janeiro, Brazil; ^2^ Instituto de Microbiologia Paulo de Góes, Universidade Federal do Rio de Janeiro, Rio de Janeiro, Brazil

**Keywords:** heteroxenous, immunocompromised hosts, monoxenic, pathogenicity, trypanosomatidae, immunocompetent host

## Abstract

The Trypanosomatidae family encompasses unicellular flagellates and obligate parasites of invertebrates, vertebrates, and plants. Trypanosomatids are traditionally divided into heteroxenous, characterized by the alternation of the life cycle between an insect vector and a plant or a vertebrate host, including humans being responsible for severe diseases; and monoxenous, which are presumably unique parasites of invertebrate hosts. Interestingly, studies reporting the occurrence of these monoxenous trypanosomatids in humans have been gradually increasing, either associated with *Leishmania* co-infection, or supposedly alone either in immunocompromised or even more sporadically in immunocompetent hosts. This review summarizes the first reports that raised the hypothesis that monoxenous trypanosomatids could be found in vertebrate hosts till the most current reports on the occurrence of *Crithidia* spp. alone in immunocompetent human patients.

## Introduction

The family Trypanosomatidae encompasses eukaryotic flagellates, unicellular and obligatory parasites of invertebrates, vertebrates, and plants. The unique morphology of its single mitochondrion DNA (called kinetoplast) is an apomorphy of the Class Kinetoplastea, and its specific cellular positioning in relation to the nucleus and the point of flagella emersion allows the identification of specific life cycle forms, some of which are genus-specific (reviewed by d’Avila-Levy et al., 2015). Trypanosomatids are traditionally divided into heteroxenous, characterized by alternating the life cycle between an insect vector and a vertebrate host or a plant; and monoxenous, which are parasites presumably exclusive of invertebrate hosts, mainly insects. The heteroxenous trypanosomatids are the causative agents of severe human diseases that are mainly transmitted by an insect vector, such as Chagas disease (caused by *Trypanosoma cruzi*), sleeping sickness (caused by *Trypanosoma brucei sensu lato*), and the various forms of cutaneous and visceral leishmaniasis (caused by *Leishmania* spp.) (Vickerman, 1994). On the contrary to these pathogenic flagellates, the monoxenous (or “lower”) trypanosomatids have received considerably less effort and attention by the scientific community since these parasites are found, a priori, only in the digestive tract of insects, and even its pathogenicity to insects is questionable, with few exceptions. Nevertheless, species typically non-pathogenic to humans are important models for understanding the biological behavior, biochemistry and molecular biology of pathogenic trypanosomatids (d’Avila-Levy et al., 2015). In addition, these organisms are being explored as vaccine candidates; for example, *Phytomonas serpens* confers protective immunity against *T. cruzi* (Pinge-Filho et al., 2005; da Silva et al., 2013); and as a platform to produce folded eukaryotic proteins, such as erythropoietin and insulin produced by *Leishmania tarentolae* (Dortay and Mueller-Roeber, 2010). Finally, monoxenous trypanosomatids are attracting the attention of researchers in the field due to the increasing reports on the occurrence of these presumably non-pathogenic trypanosomatids in humans (Dedet et al., 1995; Jiménez et al., 1996; Pacheco et al., 1998; Miller, 2000; Ferreira and Borges, 2002; Chicharro and Alvar, 2003). Here, we will present a historical review since the first reports that raised the hypothesis that monoxenous trypanosomatids could be found in vertebrate hosts till the recent reports on the occurrence of *Crithidia* spp. in immunocompetent human patients.

## First Reports on the Occurrence of Monoxenic Trypanosomatids in Humans

A comprehensive bibliographic survey on the biology and physiology of monoxenous trypanosomatids by McGhee and Cosgrove (1980) described a possible human infection attributed to the genus *Herpetomonas*. The authors reported a patient with undefined symptoms (such as fever and moments of unconsciousness) admitted to a hospital in Texas (USA). Several tests were performed, including a liver biopsy, which subsequently was inoculated in culture medium. After seven days, it was possible to observe the proliferation of flagellates. However, neither promastigote nor epimastigote forms were observed. The authors suggested that the flagellate found in the patient’s sample was a species of the genus *Herpetomonas* based on the microscopic observations and the culture behavior, but the possibility of culture contamination by the culture medium manufacturer or by sample handling in the hospital was not ruled out (McGhee and Cosgrove, 1980). In addition, this report also mentioned evidence of *Herpetomonas megaseliae* (syn. *Herpetomonas muscarum* (Borghesan et al., 2013) infection in lizards and mice (Daggett et al., 1972; McGhee and Cosgrove, 1980) and species of *Crithidia*, *Leptomonas*, and *Blastocrithidia* that managed to grow in chicken embryos maintained at 37°C (Schmittner and Mcghee, 1970). However, the authors also stated that previous claims of pathogenicity of monoxenic trypanosomatids in infection of vertebrates were made but that none of these cases could be sustained after close examination. To sum up, McGhee & Cosgrove challenged: “Although there is no proof of lower trypanosomatids infecting vertebrates, the possibility exists and should be considered by attending physicians and veterinarians” (McGhee and Cosgrove, 1980).

In 1991, an unusual clinical manifestation was reported in an immunosuppressed child in the Republic of Guinea-Bissau. The case drew physicians’ attention, as it had symptoms caused by *Leishmania* species that cause visceral leishmaniasis. However, leishmaniasis had never been reported in the country by that moment. This led the researchers to suppose that the symptoms were caused by some unknown species of *Leishmania* or another opportunistic trypanosomatid present only in reptiles or small mammals, different from the well-known pathogenic species (Sabbatani et al., 1991).

Later, in 1995, a report on the island of Martinique (France) of a human immunodeficiency virus (HIV)-positive patient who developed the diffuse cutaneous nodular syndrome, usually caused by *Leishmania* species, also caught the attention of researchers (Dedet et al., 1995). The parasites isolated from the skin lesions were submitted to isoenzymes characterization and optical and electron microscopy. Interestingly, the parasite differed isoenzymatically from all known *Leishmania* species. The authors assumed that the parasite present in the lesion could be a monoxenous trypanosomatid since both microscopies revealed the presence of the kinetoplast and opisthomastigote stages (Dedet et al., 1995), and the successful infection could be related to the immunodeficiency of the patient. Some years later, Boisseau-Garsaud et al., 2000) reported a second case, but this time in a patient not affected by any immunodeficiency, in the same location (Martinique - France) presenting a localized skin lesion. The isoenzyme analysis revealed that this parasite showed the same isoenzymatic profile of the parasite described previously by Dedet and colleagues. As none of the two patients had left the island in their entire lives, the authors suggested that an insect parasitic monoxenous trypanosomatid occurring in that region could be infecting humans in a diffuse or localized manner depending on the health status of the host (Boisseau-Garsaud et al., 2000). However, two years later, Dedet research group submitted the two clinical isolates to gene sequencing. Following the isoenzymic characterization, both strains were identical to each other and distant from any other known *Leishmania* species, although related to *L. enriettii*. Thus, it was not possible to conclude whether these isolates would be grouped within *Leishmania* (*Leishmania*), *Leishmania* (*Viannia*) or if they should belong to a new clade within the euleishmania group (Noyes et al., 2002). Several years later, these isolates were recognized as *L. martiniquensis*, with a divergent taxonomic position, distinct from any other existing taxa responsible for an endemic focus of cutaneous leishmaniasis in Martinique (Desbois et al., 2014).

In 1996, Jiménez and colleagues reported a case in Madrid of a patient who, in addition to having immunosuppression, was an injecting drug user. This patient was hospitalized with an extensive medical history of infections and, among them, clinical suspicion of visceral leishmaniasis. Although it was not possible to observe amastigote forms in the bone marrow aspirate, some flagellates were isolated in culture, and promastigote forms were observed by optical microscopy. The isoenzymatic characterization revealed a pattern different from *Leishmania* reference strains, and the sample did not hybridize with *Leishmania*-specific probes. Moreover, isolated promastigotes could not infect either BALB/c mice or Syrian golden hamsters. Therefore, the authors ruled out the possibility of *Leishmania* infection and suggested that the patient could be infected by a monoxenous trypanosomatid that could cause leishmaniasis-like symptoms in HIV-positive patients. The contamination route could be syringe washing with water contaminated with parasitic insect feces (Jiménez et al., 1996).

In Brazil, Pacheco et al. (1998) identified amastigotes in a bone marrow aspiration from an HIV-positive patient with clinical symptoms of visceral leishmaniasis. The patient’s place of residence is endemic for *L. braziliensis*, a species responsible for cutaneous leishmaniasis, which raised the hypothesis of visceralization of a typical cutaneous species. The parasite grew in culture as promastigotes and could not establish infection in mouse peritoneal macrophages, suggesting the non-pathogenicity of the flagellate (Pacheco et al., 1998). The isoenzyme analysis revealed that the isolated parasite did not correspond to all the assessed known genera, including the classical pathogenic ones. Southern blot hybridization technique revealed homology with the monoxenous species *Leptomonas pulexsimulantis* (generally parasitic species of dog fleas) (Pacheco et al., 1998). The authors assumed that the patient’s immunosuppressed condition probably facilitated the establishment of opportunistic monoxenous trypanosomatids in the organism already weakened by HIV (Pacheco et al., 1998).

At the beginning of the 2000s, the progressive increase in reports of leishmaniasis symptoms in patients caused by non-leishmania trypanosomatids prompted Chicharro and Alvar (2003) to publish the first review on the topic - Monoxenous trypanosomatids in immunosuppressed patients. Although the pathogenicity of monoxenous trypanosomatids is debatable, the authors argued that these reports are not surprising since individuals severely immunocompromised because of HIV infection may also be more susceptible to other infections, including trypanosomatids, which are generally considered non-pathogenic.

In 2008, an immunosuppressed patient was admitted to a hospital in Montpellier (France), the symptoms being fever and persistent headache. The patient had been HIV-positive since 1991 and had a history of antiretroviral failure with low CD4+ T cell counts, leading to many opportunistic diseases. Among the exams performed, a blood sample was inoculated in an NNN medium. Four weeks later, kinetoplastid flagellates with morphology different from that of typical *Leishmania* promastigotes were isolated. The patient was discharged from the hospital and recovered spontaneously without any anti-leishmanial treatment. Subsequent attempts to re-isolate the parasite either from the blood or from the bone marrow were unsuccessful. Sequencing of the *5S* and *18S* ribosomal DNA region from the isolated parasite revealed genetic proximity to the monoxenous trypanosomatid *Herpetomonas samuelpessoai* (Morio et al., 2008). It seems clear that the patient’s immune status can pave the road for opportunistic infections, like the monoxenic trypanosomatids. It is interesting to note that all these reports identified the later after culture isolation, which creates an important bias that will be later addressed.

## Co-Infections Leishmania and Leptomonas

Besides the possible infections by monoxenous trypanosomatids reported in immunosuppressed patients, another phenomenon has been attracting attention in the scientific community: the co-infection of monoxenous trypanosomatids, mainly of the genus *Leptomonas*, and *Leishmania* species. This co-infection has been reported increasingly, and most of these reports come from the Indian subcontinent, where visceral leishmaniasis or Kala-azar is frequent (Srivastava et al., 2010; Ghosh et al., 2012; Selvapandiyan et al., 2015; Thakur et al., 2020).

By screening 120 patients with kala-azar symptoms in the Indian subcontinent, Srivastava et al. (2010) obtained successful culture isolates from all splenic aspirates. The restriction fragment length polymorphisms (RFLP) of the *Hsp70* gene from the isolates identified nine samples with a restriction pattern that differed from known *Leishmania* species. Furthermore, *Hsp70* and *18S* gene sequencing of two isolates revealed a genetic relatedness to *Leptomonas seymouri* (Srivastava et al., 2010). This report is interesting because it identifies a potential monoxenic trypanosomatid in 9 different kala-azar patients from different regions of India with no history of HIV/AIDS. Although, as expected, BALB/c inoculation with *L. donovani* led to death after 20 days, while the BALB/c group inoculated with one of the atypical isolates survived and 45 days after infection, DNA was extracted, and the product of rDNA-ITS1 PCR analyzed by gel electrophoresis revealed a pattern consistent with *Lept. seymouri* and *L. donovani* (Srivastava et al., 2010). This raised concerns on the original culture: was it mixed with a higher proportion of *Lept. seymouri* that outnumbers but does not eliminate *L. donovani* in culture? Kala-azar causes a significant immunosuppression that could pave the road for *Lept. seymouri* infection; therefore, in mice, the few *L. donovani* cells would increase, but the inoculum would not be enough to establish a deadly infection. Two years later, another research group showed that *Lept. seymouri* was identified in four out of 29 DNA clinical samples (peripheral blood or skin biopsy) from patients with visceral leishmaniasis or post-kala-azar dermal leishmaniasis (PKDL). In addition, two out of seven parasites isolated in culture from blood samples revealed similarity to *Lept. seymouri* (Ghosh et al., 2012), which was first identified due to an atypical ITS1 PCR product, followed by DNA sequencing and phylogenetic analysis (Ghosh et al., 2012). Later, a screening by ITS1-RFLP of cutaneous leishmaniasis biopsies from 57 patients in a new endemic region in India revealed that 38.5% (22/57) of the samples presented two PCR products with a restriction fragment profile consistent with *L. donovani* and *Lept. seymouri*. Subsequently, the amplicons from representative samples (n=9) of the possible co-infection biopsies were sequenced, revealing maximum identity with *L. donovani* and *Lept. seymouri* (Thakur et al., 2020). Considering, that the PCR was performed directly in the biopsies, this is an important piece of evidence towards the participation of monoxenic trypanosomatids in leishmaniasis physiopathology.

Usually, when patients manifest the typical clinical features of visceral leishmaniasis, PKDL or cutaneous leishmaniasis, the test performed to confirm the diagnosis is either the detection of the parasite in smears on a stained slide or culture positivity; in some cases, ELISA tests are also used. However, DNA sequencing is rarely performed to confirm the identity of the species found. This has led the authors to question whether all the parasites isolated from these patients with leishmaniasis may be infected with *L. donovani* and any potential co-infecting monoxenous trypanosomatid species, since they are not typed (Srivastava et al., 2010; Thakur et al., 2020). Interestingly, the authors stated that the detection of the non-pathogenic species in patients in the Indian sub-continent must be carefully considered: first, a more in-depth study on the clinical relevance and susceptibility of this parasite to infect humans is worth; second, considering that the co-infections were identified in an area where there are a relevant number of patients resistant to the antimonial used for the treatment of visceral leishmaniasis, this strain of *Leptomonas* could be more resistant to antimony or could act synergistically to *L. donovani* leading to treatment resistance (Ghosh et al., 2012).

In this same direction, Singh et al. (2013) using the next generation sequencing of samples collected from patients affected by visceral leishmaniasis in India, identified *Lept. seymouri* in co-infection with *L. donovani*. Although both species were in concomitance in splenic aspirates, it was not possible to attribute the pathogenicity of *Leptomonas* to the clinical cases. Furthermore, the two species have low genetic divergence and are morphologically similar: both have promastigote forms within the host insect. Thus, the researchers also believe that cases like this are underestimated and that the presence of *Leptomonas* or another monoxenous associated with the *Leishmania* species is greater than reported (Singh et al., 2013).

To evaluate the mechanisms that may favor the appearance of monoxenous trypanosomatids in vertebrate hosts, Kraeva et al. (2015) further investigated two of the clinical isolates of *Lept. seymouri* found in Singh’s work (Singh et al., 2013). Although the isolates proved to be adapted to grow at high temperatures and they were able to remain in the digestive tract of the insect vector, *Lept. seymouri* was unable to establish an infection in mammalian macrophages *in vitro*. The authors concluded that despite the adaptations undergone by *Lept. seymouri*, its occurrence in a vertebrate host would only be possible under host immunosuppressant conditions. Those authors also point out that some DNA sequences deposited at Genbank as *L*. *donovani* are in fact *Lept. seymouri* and emphasized the importance of specific markers for identifying monoxenous species (Kraeva et al., 2015).

## Crithidia Occurrence in Immunocompetent Patients

In 2019, Ghobakhloo and colleagues reported the occurrence of *Crithidia* either alone or in co-infection with *L. major* in human patients in Iran with no apparent immunosuppression. As expected, out of 167 patients screened, 92.8% had *L. major* as the only causative agent, while 5.4% accounted for co-infections, and in only 1.8% of the cases (4 patients), *Crithidia* was the sole agent. The study was focused on detecting insect trypanosomatids based on previous reports in Iran that provided preliminary evidence on the occurrence of insect trypanosomatids in immunocompetent patients. Considering the skepticism of the scientists on the occurrence of “monoxenous” trypanosomatids in healthy individuals and the general belief that all reports up to date are due to the culture strain/clone biased selection, the authors collected samples directly from the biopsies and performed microscopy and PCR amplification. Using two sets of gGAPDH primers, one that amplified *Leishmania* and the other *Crithidia*, the authors provided sequences that showed similarity to *C. fasciculata* reference sequences (Ghobakhloo et al., 2019). In addition to the molecular characterization of the clinical samples, the authors also analyzed some aspects of the isolates obtained in culture. The isolates were able to survive, even if less active and rounder, at a higher temperature that mimicked the temperature of the human body, in contrast to a *C. fasciculata* reference strain obtained from the Centre of Research in Infectious Disease, Laval University (Quebec, Canada). In addition, the clinical isolate of *Crithidia* was able to infect two macrophage cell lines (J774 and THP1). However, as expected, the infection index was lower when compared to *L*. *infantum* and *L. major* infection. Furthermore, the reference strain of *Crithidia* did not show the same behavior, it was unable to establish itself inside the macrophages (Ghobakhloo et al., 2019).

These reports open interesting discussions: for a time, the findings of monoxenous trypanosomatids in humans were associated with immunodeficiencies in the patient. In this work, some isolates of *Crithidia* were recovered from the lesions of immunocompetent patients. In addition, when *Leishmania*-*Crithidia* co-infection was reported, the clinical cases were more severe when compared to patients who had only *Leishmania* isolated from the lesion. Another important observation is that, as described in the co-infection between *Lept. seymouri* and *L. donovani* (Ghosh et al., 2012), some of these co-infections did not respond to treatment with antimonial, which raised the question of the possible role of monoxenous trypanosomatids in resistance to the therapy (Ghobakhloo et al., 2019).

This article was refuted in the same year by Kostygov et al., 2019) who claimed that the methodologies used in Ghobakhloo’s report were not sufficient to state that *Crithidia* was the sole agent causing skin damage in immunocompetent patients. Kostygov and colleagues claimed that the immune status of the patients was not assessed as well as the primer used to amplify gGAPDH from *Leishmania* could not amplify *L. infantum* (Kostygov et al., 2019). Subsequently, the authors replied that the *Crithidia* spp. were identified in patients with cutaneous leishmaniasis resistant to glucantime treatment, which led the researchers to a closer follow up of these patients. To this end, all patients were examined for immunodeficiency diseases, which were excluded, and then biopsies and smears from patients’ lesions were collected under sterile conditions for parasite characterization. One sample was taken for culture, and three smears were prepared for microscopic study and PCR amplification. Regarding the PCR primer specificity, the authors replied that it could discriminate among *L. infantum, L. major*, and *L. tropica* (Motazedian, 2019). Concerning the specificity of the PCR primer designed to amplify *Leishmania*, the primer specific for *Crithidia* did amplify a fragment directly from the patients’ smears. The amplicon was sequenced and revealed similarity to *Crithidia*. The same sample revealed only *Crithidia* in the cultures, subsequently identified in Dr. Marc Ouellette’s research group (Laval University Research Center for Infectious Diseases, Quebec, Canada). Kostygov hypothesis would be that these four samples would be a co-infection between *L. infantum* and *Crithidia*, and the authors failed to detect the former by the PCR primers used. Then, as expected, in culture, the monoxenic counterpart outgrows the fastidious *Leishmania*.

In the same year, another report drew a lot of attention from the scientific community, also reaching the general press (Maruyama et al., 2019). The authors described one fatal case of a visceral leishmaniasis-like, being more severe and resistant to the treatment. The patient, which had a negative HIV diagnosis, died of disease and surgical complications. The parasite was isolated in culture from skin lesion and bone marrow. The isolated parasite is more closely related to *C. fasciculata* than to any *Leishmania* by several approaches, including whole-genome sequencing. After intravenous infection in female BALB/c mice with both isolates, only the bone marrow isolate was detected again in the liver and spleen; however, at significantly lower levels than the positive control (*L. infantum*), while the skin strain was detected at very low levels only in the liver. Therefore, those authors performed an artificial skin infection on the mice’s ear, revealing that the skin strain caused lesions even more extensive than that resulting from the positive control (*L. major*) (Maruyama et al., 2019). Subsequently, Domagalska and Dujardin published a comment letter where they raised concerns to what we can call – “environment biased selection hypothesis”. The inoculation in the culture medium from the patient’s biopsy can lead to the monoxenic counterpart prevailing in culture, although few *Leishmania* cells can remain. After that, in the mouse, the few *Leishmania* cells can outnumber the *Crithidia*. Therefore, a molecular identification after the experimental infection can help solve this puzzle (Domagalska and Dujardin, 2020). Finally, the last report sequencing the *ITS1* gene directly from buffy coat samples in 14 patients (seven symptomatic and seven asymptomatic) in Iran revealed that one asymptomatic sample presented close similarity (99.75%) to *C. fasciulata* (Rezaei et al., 2020).

## Occurrence of “Monoxenous” Trypanosomatids in Non-Human Mammalian Hosts

Human infection possibly caused by insect trypanosomatids calls the attention of the scientific community from the first report. However, not only humans are reported as possible unusual mammalian hosts for these trypanosomatids (Maslov et al., 2013). The possibility that insect trypanosomatids could be found in mammalian hosts started in the early description of these parasites, already by morphological observations (McGhee and Cosgrove, 1980). The first time, to our knowledge, that a DNA sequence was provided from monoxenic trypanosomatids isolated from mammals was from rodents and dogs in Egypt several years after the original isolation. The original parasite kept in culture for several years was sequenced and reported to be close to *Herpetomonas* (Podlipaev et al., 2004). A long jump in years and a more recent report performed DNA sequencing screening on 593 insectivorous bats from 8 species in the USA, revealing that 5 (0.8%) were positive for *Blastocrithidia* spp. The incidence is not to be neglected since the positivity for *T. cruzi*, and *T. dionisii* were 0.17% and 1.5%, respectively (Hodo et al., 2016). The screening was performed on DNA extracted from blood and heart tissue with no culture manipulation (Hodo et al., 2016). Another bat screening performed in Brazil revealed that out of 181 bat specimens from 18 species, one was found positive for a species belonging to the *Crithidia* genus, most likely *C. mellificae* (Rangel et al., 2019). The DNA amplification and sequencing were performed out of a culture isolate after rich medium cultivation (Rangel et al., 2019). In a following report from the same research group, a screening in 72 sylvatic animals captured in three distinct biomes in Brazil (Atlantic Forest, Cerrado, and Pantanal) revealed choanomastigote forms in 21 fresh hemoculture preparations (from marmosets, coatis, crab-eating fox, and ocelots). Parasites could not be observed in direct blood examination (Dario et al., 2021). DNA extraction from these hemocultures and *18S* and *gGAPDH* genes sequencing revealed a close similarity with *C. mellificae* (Dario et al., 2021).

The description of natural infections of monoxenous trypanosomatids in mammals has a tendency to increase. However, the actual parasite-host relationship is not fully established, not even if it is a parasite as classically defined. There are though several reports demonstrating successful *in vitro* infections by monoxenous trypanosomatids in humans and mouse cellular culture, as well as the successful growth of these parasites at 37 °C (Santos et al., 2004; Barreto-de-Souza et al., 2008; Matteoli et al., 2009; Pereira et al., 2010).

## Final Considerations

The first report of a monoxenous trypanosomatid causing disease in mammals occurred as early as the 1980’s. The reports are increasing in number and consistency. One should consider that for the general physician routine and patient handling and treatment, parasite isolation and identification is not necessary, and it is rarely performed. Therefore, the cases that resulted in a scientific report were either because *Leishmania* was not an occurring pathogen in the country or because the physicians’ faced hurdles in the patient treatment, which required an “out of the box” approach. Anyway, the routine leishmaniasis diagnosis can never reveal the occurrence of monoxenic trypanosomatids: direct smears observation and immunoassays can reveal positivity for a monoxenic trypanosomatid without distinguishing from *Leishmania*. Isoenzymes and DNA sequencing can reveal the identity of the pathogen. However, the former requires parasite isolation in culture, while the latter can be performed directly in the patients’ biopsy, but it is not frequently performed. Although it seems unequivocal that monoxenic trypanosomatids are occurring in wild mammals and humans and even in immunocompetent ones, the “environment biased selection hypothesis” is far from ruled out. Therefore, a co-infection monoxenic trypanosomatids/*Leishmania* can be more common than imagined; the subsequent culture inoculation can favor the fast-growing monoxenic counterpart, which outgrows *Leishmania* without completely eliminating it from the culture media. Then, a mice infection can promote the selection in the other direction. Therefore, in order to take steps ahead on this intriguing pathway that could completely change our understanding of the trypanosomatids life cycle, it is critical that DNA amplification and sequencing is performed in the original biopsy, in the cultured sample, and then upon parasite isolated after the experimental infection. Nevertheless, it is time to move forward. The occurrence of monoxenic trypanosomatids in mammals is still viewed with suspicion by the scientific community. Several published reports needed to justify that there was no misidentification or contamination during the handling of the material, and primer specificity is questioned. Extensive screening of clinical samples by direct DNA sequencing would shed some light on the frequency of monoxenic trypanosomatids in humans and, more importantly, provide further evidence on their relevance in treatment resistance. Subsequently, further questions arise: how do they reach the lesion? As a secondary infection by a fly or as direct inoculation by a vector? The questions that arise are endless, but the first and urgent approach is – are they alone or in a co-infection with *Leishmania*? A detailed study using the powerful DNA sequencing tools available today may provide a significant increase in knowledge about the possible role of monoxenous trypanosomatids in the pathogenic process, either alone or in co-infection with traditional pathogenic species.

## Author Contributions

CMDL and CBM wrote the first version of the manuscript. All the authors discussed, conceived and revised the manuscript. All authors contributed to the article and approved the submitted version.

## Funding

We thank the Brazilian grant agencies Coordenação de Aperfeiçoamento de Pessoal de Nível Superior (CAPES), Conselho Nacional de Desenvolvimento Científico e Tecnológico (MCT/CNPq), Fundação Carlos Chagas Filho de Amparo à Pesquisa do Estado do Rio de Janeiro (FAPERJ) for funding, as well as Fundação Oswaldo Cruz (FIOCRUZ) for research infrastructure.

## Conflict of Interest

The authors declare that the research was conducted in the absence of any commercial or financial relationships that could be construed as a potential conflict of interest.

## Publisher’s Note

All claims expressed in this article are solely those of the authors and do not necessarily represent those of their affiliated organizations, or those of the publisher, the editors and the reviewers. Any product that may be evaluated in this article, or claim that may be made by its manufacturer, is not guaranteed or endorsed by the publisher.
